# Sialidase Activity in Human Blood Serum Has a Distinct Seasonal Pattern: A Pilot Study

**DOI:** 10.3390/biology9080184

**Published:** 2020-07-22

**Authors:** Victor Y. Glanz, Dmitry A. Kashirskikh, Andrey V. Grechko, Shaw-Fang Yet, Igor A. Sobenin, Alexander N. Orekhov

**Affiliations:** 1 Institute of Human Morphology, 3 Tsyurupa Street, 117418 Moscow, Russia; viglanz@outlook.com (V.Y.G.); dim.kashirsckih@gmail.com (D.A.K.); igor.sobenin@gmail.com (I.A.S.); 2 Institute of General Pathology and Pathophysiology, 8, Baltiyskaya st., 125315 Moscow, Russia; 3 Federal Research and Clinical Center of Intensive Care Medicine and Rehabilitology, 14-3 Solyanka Street, 109240 Moscow, Russia; noo@fnkcrr.ru; 4 Institute of Cellular and System Medicine, National Health Research Institutes, 35 Keyan Road, Zhunan Town, Miaoli County 35053, Taiwan; syet@nhri.org.tw; 5 Laboratory of Medical Genetics, National Medical Research Center of Cardiology, 15A 3-rd Cherepkovskaya Street, 121552 Moscow, Russia; 6 Institute for Atherosclerosis Research, Skolkovo Innovative Center, 121609 Moscow, Russia

**Keywords:** sialidase, atherosclerosis, influenza

## Abstract

Desialylation—loss of terminal sialic acid residues from glycoconjugates catalyzed by sialidases—is involved in many human diseases and is considered a key molecular event of atherosclerosis onset. Desialylated low-density lipoproteins with atherogenic properties have been detected in human blood previously. However, there is currently no consensus on the origin of desialylation activity in the bloodstream. Here, we suggest viral intervention as a possible explanation. In order to address our hypothesis, we studied seasonal patterns of blood serum sialidase enzymatic activity and designed an approach to detect and quantify viral sialidase genetic presence. Increased sialidase activity in autumn-winter combined with detectable levels of influenza virus sialidase mRNA suggests exogenous viral sialidase as a viable component of desialylation in human blood, providing new insights on the molecular background of atherogenesis.

## 1. Introduction

Desialylation is an enzymatically aided process of sialic acid removal/transfer from glycocalyx moieties, which is performed by several classes of enzymes: sialidases (neuraminidases), sialyltransferases and trans-sialidases. Sialidases, which are exoglycosidases found in vertebrates, protists, bacteria and viruses, are known to be involved in the pathogenesis of various human diseases [1,2,3]. In mammals, four sialidases are known to be encoded by NEU1, NEU2, NEU3 and NEU4 genes. It is well established that low-density lipoprotein (LDL) causes lipid accumulation in the arterial walls that primes atherogenesis [4,5]. However, the key role in this process belongs to atherogenetically modified rather than native LDL [6]. The key LDL modification is desialylation of apoprotein polysaccharide chains, which renders LDL particles atherogenic and prone to subsequent modification, such as oxidation and aggregate formation. Circulating desialylated LDL has been previously detected in human blood and described as pathologic, causing cholesterol accumulation in the arterial wall cells ultimately leading to atherosclerosis development [7]. Formation of desialylated LDL in the blood plasma is aided by the sialidase activity, which has been detected and measured in previous studies performed by our group [8,9]. Desialylation in human plasma was reported by a number of studies in several diseases, including cancer [10,11,12,13,14,15]. However, the origin of pathology-associated desialylation in human blood plasma remains a matter of speculation. Considering that sialidase plays an important role in the development of viral infection facilitating influenza virus replication [16,17], here we test the hypothesis of blood desialylation viral origin by evaluating seasonal changes in the enzymatic activity. At the same time, we attempted to establish a method for detection and quantification of exogenous sialidase mRNA by qPCR. We found that sialidase activity peaked during autumn–winter period, and in certain samples it correlated with detectable levels of influenza virus sialidase mRNA, thus supporting our hypothesis.

## 2. Materials and Methods

### 2.1. Blood Serum Collection

Blood samples were collected from apparently healthy volunteers recruited from the visitors’ flow at the municipal outpatient clinics in Moscow (Russian Federation), who had passed a routine medical examination within the frame of regular health monitoring. The study enrolled 23 participants (12 men, 11 women) who had provided an informed consent to participate in the study and were further followed-up for 6 months. The presence of clinical signs of acute respiratory diseases and influenza within 2 weeks prior to examination was the exclusion criterion. Fasting blood samples were collected in December and July. The mean age of study participants was 53.6 years (SD = 12.2); total cholesterol was 219 mg/dL (SD = 46); HDL cholesterol was 63 mg/dL (SD = 14); LDL cholesterol was 122 mg/dL (SD = 42); plasma triglycerides were 121 mg/dL (SD = 42); fasting blood glucose was 4.9 mL/L (SD = 0.9); body mass index was 25.5 kg/m^2^ (SD = 4.1). There were no statistically significant differences in biochemical characteristics between blood samples taken in winter and summer.

Cellular fraction was obtained by centrifuging for 20 min at 1600× *g* on Beckman GPR centrifuge. Serum was aliquoted and stored at −70 °C.

### 2.2. Sialic Acid Labeling with Fetuin

An equal volume of 0.1-M acetate buffer (pH 4.0) was added to 250 μL of fetuin solution (2 mg/mL). Reaction mix was then cooled to 4 °C, placed on ice, and 50 μL of freshly prepared 10-mM NaIO4 water solution was added. The mix was incubated for 10 min while being constantly shaken. The reaction was stopped by adding 50 μL of glycerin followed by 10 min-long incubation under the same conditions. Obtained solution was dialyzed against 2000 volumes of phosphate–saline (рН 7.0) at 4 °C changed twice per day. Ten microliters of NaB[3 H] 4 in 0.1-М NaOH was added to oxidized substrate-containing solution at room temperature. Samples were then incubated for 30 min at 20 °C being shaken constantly. Ten microliters of 0.1-M unlabeled NaBH4 was then added and the whole mix was incubated for 30 min. The samples were dialyzed for 48 h against 2000 volumes of phosphate–saline (рН 7.0) changed 4 times during dialysis. Obtained labeled fetuin was sterilized by filtration through 450 nm filter.

### 2.3. Preparation of Agarose Gel Covalently Linked to Fetuin Labeled with Sialic Acid Tritium

A total of 300 μg of dry bromine cyan-activated agarose was resuspended in 15 mL of 1-mM HCl (рН 4.5) and then incubated for 1 h with constant shaking. The gel was centrifuged for 10 min at 3150× *g* and supernatant was removed, followed by washing of the sediment with 15 mL of 1-mM HCl, and then the gel was centrifuged again. The sediment was washed with 15 mL of 0.2-M carbonate–bicarbonate buffer (pH 8.5), then, after centrifugation, supernatant was removed. Five milliliters of fetuin labeled with sialic acid tritium solution (0.4 mg/mL) was added to activated agarose. The samples were incubated for 2 h at room temperature under constant shaking. The suspension was then centrifuged for 10 min at 3150× *g*, and supernatant was removed. Fifteen milliliters of 20-mM glycine buffer (pH 8.5) was added to the sediment to block unreacted CN-residues, and the mixture was incubated for 1 h. The resulting gel was washed 5 times with 20 mL of 0.2-M acetate (pH 4.5) and 0.2-M carbonate–bicarbonate (pH 8.5) buffers. Finally, 2 mL of 50-mM Tris-HCl (рН 7.0) was added and the gel was stored at 4 °C.

### 2.4. Acidic Hydrolysis for Obtaining of Desialylated Fetuin

One milliliter of 0.1-N H2SO4 was added to 5 mg of protein. The mixture was incubated at 80 °C for 1 h in a water bath, after that protein solution was neutralized with 0.1-N NaOH to pH 7.0. The samples were dialyzed against phosphate–saline (pH 7.0) at 4 °C overnight and sterilized by filtration as described before.

### 2.5. Measurement of Sialidase Activity with Labeled with Sialic Acid Tritium Fetuin, Covalently Linked to Agarose Gel, as a Substrate

60 μL of labeled fetuin (sialic acid donor) linked with agarose suspension were added to 200 μL of serum. Measurement was performed in two variations: with addition of 30 μL of desialylated fetuin (sialic acid acceptor) and without. Reaction mix volume was adjusted to 300 μL with 50-mM Tris-HCl (рН 7.0) and then incubated at 37 °C for 3 h in the dark with constant shaking. After this, 200 μL of water were added and the mixture was centrifuged for 10 min at 3150× *g*. Two hundred microliters of supernatant were transferred into liquid scintillation counting containers with 5 mL scintillation (ZhS-8, Reakhim, Kharkiv, Ukraine) and a number of tritium beta decays was measured on 1215 Rack-Beta counter (LKB, Bromma, Sweden).

### 2.6. Molecular Genetics Experiments

Total RNA was extracted from blood with ExtractRNA reagent (Evrogen, Moscow, Russia) and quantified on U-2900 spectrophotometer (Hitachi, Japan). One microgram of total RNA was used as a template for reverse transcription (RT) with MMLV RT kit (Evrogen, Moscow, Russia). Endogenous (human) transcripts were converted into cDNA using oligo (dT) primers; for exogenous (viral) transcripts specific primer was used (see the sequence of viral RT primer in Table 1). Primer annealing was done at 70 °C for 5 min, cDNA first strand synthesis at 37 °C for 30 min and reverse transcription inactivation with EDTA at 65 °C for 10 min. qPCR was done on CFX96 Touch real-time PCR detection system Bio-Rad, USA) with qPCRmix-HS master mix (Evrogen, Moscow, Russia). Primers used qPCR are listed in Table 1. CAP1 and GAPDH were used as housekeeping genes in all cases, however, no specific viral gene could be used as a virus-specific normalization control.

## 3. Results

We studied enzymatic trans-sialidase activity in blood serum samples during summer and winter periods. Samples incubated without sialic acid acceptor were an indication of sialidase activity, while samples incubated with desialylated fetuin indicated combined sialidase and trans-sialidase activity. We used the difference in percentage of labeled sialic acid transferred from the gel into a solution between samples with sialic acid acceptor and samples without sialic acid acceptor as a way to represent trans-sialidase activity in serum.

**Figure 1:**
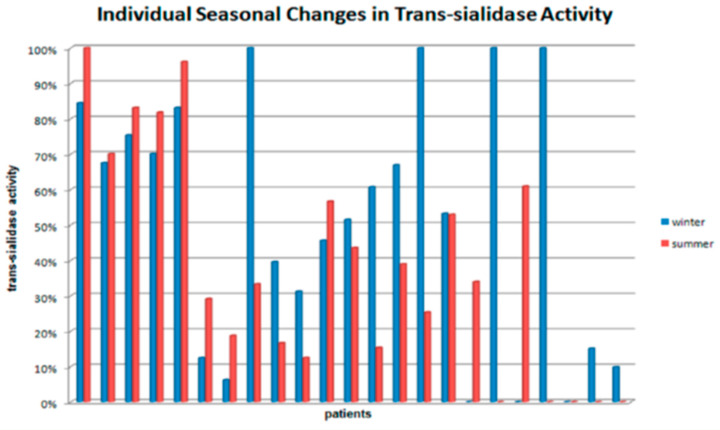
Seasonal dynamics of sialidase activity in blood serum of study participants.

When analyzing seasonal changes in trans-sialidase activity in the blood serum, the increase by more than 20% in one of the seasons could be considered reliable. Based on this criterion, we distinguished three subsets of volunteers: (1) the ones who did not display any change in trans-sialidase activity during the year, (2) the ones with reliable activity increase in winter period, (3) the ones with reliable activity increase in summer period (Figure 1). Almost 39% of volunteers demonstrated no significant change in trans-sialidase seasonal activity. However, among the rest, the number of individuals with trans-sialidase activity in serum peaked in winter was almost 3 times larger than the number of those with the opposite dynamics (43% and 17%, accordingly, of total number of volunteers).

**Figure 2:**
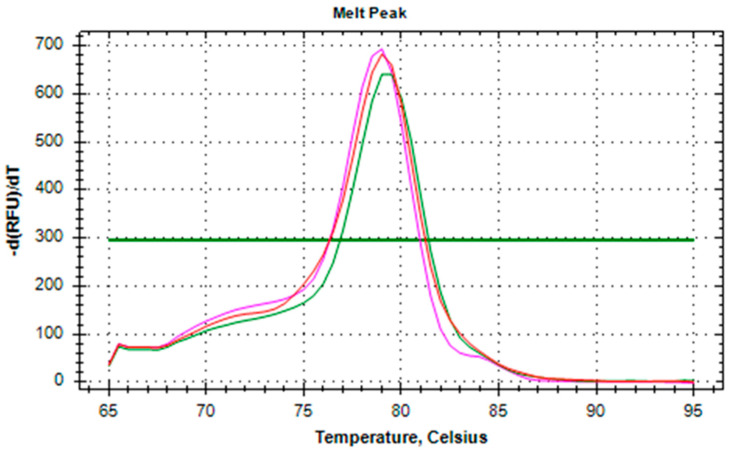
Specificity of qPCR analysis of exogenous sialidase expression indicated by single melt peak after thermal denaturation of amplicons with SYBR Green I.

To assess the contribution of exogenous sialidase, we designed a series of molecular genetic experiments and performed a pilot study. We implemented double discrimination (at RT and qPCR steps) to ensure the specificity of the analysis. The use of specific RT primer (with complementarity to highly conservative regions of sialidase-encoding genome regions) allowed only influenza virus RNA to be converted into cDNA when using total blood RNA as a starting material. Therefore, if no fluorescent signal was detected by qPCR, then there was no viral sialidase activity present. Positive result would indicate specific presence of influenza virus sialidase mRNA. Primers used for quantification of exogenous sialidase mRNA were designed to represent total activity of sialidase of several most studied influenza viral strains. Initial analysis of 6 blood samples showed that 3 of them were positive for exogenous sialidase mRNA expression with Cq values ranging from 34 to 36. Endogenous sialidase mRNA for NEU1-4 genes was detected in all samples. To estimate the specificity of exogenous sialidase expression results we performed qPCR products melting analysis within the temperature range from 65.0°C to 95.0 °C with 0.5 °C increment using host genes CAP1 and GAPDH as housekeeping controls for both host and viral genes. The results of the expression analysis of endogenous sialidases (NEU1-4) and exogenous sialidase (influenza virus neuraminidase) are presented in Table 2. Visualization with CFX Manager Software revealed a single symmetric melt peak for all studied reactions indicating the presence of only one amplicon with no background nonspecific amplification (Figure 2). The analysis of NEU1-4 genes is planned for future studies by our group.

Initial results of the genetic study obtained using the described method appear to be promising for conducting future larger-scale experiments.

## 4. Discussion

Seasonal changes can be observed for various biochemical parameters measured in blood, such as hemoglobin concentration, blood viscosity, cell fractions ratio, as well as activity and levels of certain serum and intracellular enzymes [18]. In general, these changes indicate stress-induced response under various environmental or social factors (low temperatures, sedentary lifestyle, short light day, etc.). Thus, elevated blood serum trans-sialidase activity during winter in significant part of population may serve as an additional risk factor for cardiovascular diseases increased frequency during the autumn–winter period. Suggested direct effect of trans-sialidase on LDL in the blood may lead to the development and progression of atherosclerotic lesions during the periods of increased blood trans-sialidase activity. In this work, we assessed the season-dependent variability of sialidase activity in humans for the first time.

The study had certain limitations. First, the study was designed a pilot one and had a small sample size. The results delivered by the study can therefore be considered as preliminary. The obtained results should be further confirmed and expanded in future studies. Second, the flu often happening in autumn and winter season should certainly have an effect on plasma sialidase activity. In our study, the survey of study participants performed in July showed that only four of them had a flu during the winter season between the two medical examinations. Such small proportion did not allow performing a valid correlation analysis of association of sialidase activity and flu infection. Moreover, we cannot exclude the possibility of subclinical cases of flu, as well as of the contacts with infected subjects. It would be interesting to test the possibility of increased desialylation activity in the blood plasma of patients with and without confirmed viral infection on a population, which is sufficiently large to assess possible changes. Alternatively, changes of plasma tarns-sialidase activity should be measured at different times through the year to assess possible seasonality, which would indirectly indicate a link with flu infection. Moreover, data obtained using qPCR should be reinforced by the analysis of protein expression. These questions will be clarified by future studies. Third, this pilot study was performed using non-normalized assessment of target gene expression. These experiments should be repeated using robust controls to confirm the preliminary results. Finally, since the possibility of affecting platelets clearance by desialylation exists [19], platelet counts may serve as indirect indicator of increased sialidase activity. Our study was performed within the frame of health monitoring program, which did not include platelet count.

In spite of the above limitations, according to the results of the initial stage of genetic experiments, the pattern of seasonal change in sialidase activity can be explained, at least partially, by contribution of background viral infection, of sialidase expressed by influenza virus, supporting the suggestion that sialidase activity in human blood is a combined contribution of endogenous and exogenous activities.

## 5. Conclusions

We studied seasonal changes in desialylation activity in human blood serum and demonstrated a pattern of increased sialidase activity during the autumn–winter period. This period is also associated with increased susceptibility and presence of viral infections in the population, so we suggested the viral contribution to the origin of increased desialylation in the blood and developed an approach to detect and quantify influenza virus sialidase mRNA. Pilot study demonstrated viability of the proposed method, and initial data confirmed our hypothesis. However, further research is needed to extend our study, which may help gaining important insights about the origin of sialidase activity in human blood.

## Figures and Tables

**Table 1:** Primers used for qPCR.

Primer	Sequence (5′-3′)
NEU1	f-CGCTACGGAAGTGGGGTCAG r-AGTCCTGAAGGCAGAATACC
NEU2	f-ACCAGGTTCAGTGGCAAGCTC r-GTGAAGTTTCCGGTAGGCGT
NEU3	f-CAGTGCAGAGGTCATGGAAGAA r-AAGTCCCTCACCTCACTCCA
NEU4	f-CCTTCACGGACAGTGCTCTT r-AATGTGGCCCAGTCCTGC
Viral RT primer	AGCAAAAGCAGG
Viral sialidase	f-TATTGGTCTCAGGGAGCAAAAGCAGGAGT r-ATATGGTCTCGTATTAGTAGAAACAAGGAGTTTTTT

**Table 2:** Relative expression of endogenous and exogenous sialidases (presented as mean values with standard deviations).

Type of Genetic Target	Expression of Endogenous and Viral Sialidases				Р (ANOVA)
	Total *n* = 23	Low Sialidase Activity *n* = 11	Moderate Sialidase *n* = 5	High Sialidase Activity *n* = 7	
*NEU1*	0.3 (1.0)	0.3 (0.7)	0.0 (0.0)	0.6 (1.5)	0.45
*NEU2*	6.3 (12.6)	5.4 (10.9)	1.1 (1.4)	10.9 (17.3)	0.21
*NEU3*	0.7 (2.6)	1.2 (3.8)	0.1 (0.1)	0.3 (0.6)	0.50
*NEU4*	0.3 (0.6)	0.3 (0.6)	0.2 (0.3)	0.4 (0.9)	0.80
Exogenous *NEU*	0.5 (1.0)	0.4 (0.8)	0.8 (1.3)	0.4 (0.9)	0.59
